# Correction: SHP-1 regulates hematopoietic stem cell quiescence by coordinating TGF-β signaling

**DOI:** 10.1084/jem.2017147706252018c

**Published:** 2018-08-06

**Authors:** Linjia Jiang, Xue Han, Jin Wang, Chen Wang, Xiaoqiang Sun, Jiayi Xie, Guojin Wu, Hiep Phan, Zhenguo Liu, Edward T.H. Yeh, ChengCheng Zhang, Meng Zhao, Xunlei Kang

Vol. 215, No. 5, May 7, 2018. 10.1084/jem.20171477.

The authors regret that Edward T.H. Yeh was inadvertently left off the original author list. The corrected author list appears above. The online article has been corrected accordingly. The error exists only in the print version.

